# Optically Guided High-Frequency Ultrasound to Differentiate High-Risk Basal Cell Carcinoma Subtypes: A Single-Centre Prospective Study

**DOI:** 10.3390/jcm12216910

**Published:** 2023-11-03

**Authors:** Szabolcs Bozsányi, Mehdi Boostani, Klára Farkas, Phyllida Hamilton-Meikle, Noémi Nóra Varga, Boglárka Szabó, Flóra Vasanits, Enikő Kuroli, Fanni Adél Meznerics, Kende Lőrincz, Péter Holló, András Bánvölgyi, Norbert M. Wikonkál, Gyorgy Paragh, Norbert Kiss

**Affiliations:** 1 Department of Dermatology, Venereology and Dermatooncology, Semmelweis University, 1085 Budapest, Hungary; szabolcs.bozsanyi@roswellpark.org (S.B.); mehdi_parsii@yahoo.com (M.B.); farkas.klara@phd.semmelweis.hu (K.F.); pkhmeikle@gmail.com (P.H.-M.); varga.noemi@stud.semmelweis.hu (N.N.V.); boglarka1.szabo@gmail.com (B.S.); vasanits.flora@stud.semmelweis.hu (F.V.); kuroli.eniko@med.semmelweis-univ.hu (E.K.); meznerics.fanni@stud.semmelweis.hu (F.A.M.); lorincz.kende@med.semmelweis-univ.hu (K.L.); hollo.peter@med.semmelweis-univ.hu (P.H.); banvolgyi.andras@med.semmelweis-univ.hu (A.B.); wikonkal.norbert@med.semmelweis-univ.hu (N.M.W.); 2 Department of Dermatology, Roswell Park Comprehensive Cancer Center, Buffalo, NY 14203, USA; gyorgy.paragh@roswellpark.org

**Keywords:** basal cell carcinoma, high-frequency ultrasound, histological subtype, dermoscopy, aggressive subtype, non-melanoma skin cancer, biopsy, early detection, surgical excision

## Abstract

Background: Basal cell carcinoma (BCC) is the most common type of skin cancer in the Caucasian population. Currently, invasive biopsy is the only way of establishing the histological subtype (HST) that determines the treatment options. Our study aimed to evaluate whether optically guided high-frequency ultrasound (OG-HFUS) imaging could differentiate aggressive HST BCCs from low-risk tumors. Methods: We conducted prospective clinical and dermoscopic examinations of BCCs, followed by 33 MHz OG-HFUS imaging, surgical excision, and a histological analysis. We enrolled 75 patients with 78 BCCs. In total, 63 BCCs were utilized to establish a novel OG-HFUS risk classification algorithm, while 15 were employed for the validation of this algorithm. The mean age of the patients was 72.9 ± 11.2 years. Histology identified 16 lesions as aggressive HST (infiltrative or micronodular subtypes) and 47 as low-risk HST (superficial or nodular subtypes). To assess the data, we used a one-sided Fisher’s exact test for a categorical analysis and a Receiver Operating Characteristic (ROC) curve analysis to evaluate the diagnostic accuracy. Results: OG-HFUS distinguished aggressive BCC HSTs by their irregular shape (*p* < 0.0001), ill-defined margins (*p* < 0.0001), and non-homogeneous internal echoes (*p* = 0.004). We developed a risk-categorizing algorithm that differentiated aggressive HSTs from low-risk HSTs with a higher sensitivity (82.4%) and specificity (91.3%) than a combined macroscopic and dermoscopic evaluation (sensitivity: 40.1% and specificity: 73.1%). The positive and negative predictive values (PPV and NPV, respectively) for dermoscopy were 30.2% and 76.8%, respectively. In comparison, the OG-HFUS-based algorithm demonstrated a PPV of 94.7% and an NPV of 78.6%. We verified the algorithm using an independent image set, *n* = 15, including 12 low-risk and 3 high-risk (high-risk) with two blinded evaluators, where we found a sensitivity of 83.33% and specificity of 91.66%. Conclusions: Our study shows that OG-HFUS can identify aggressive BCC HSTs based on easily identifiable morphological parameters, supporting early therapeutic decision making.

## 1. Introduction

Basal cell carcinoma (BCC) is the most common type of cancer in the Caucasian population [1,2]. It is a non-melanoma skin cancer with a very low metastatic potential [3], but it can be aggressive and locally destructive [4]. BCCs are located mainly in the head and neck area (around 80% of all cases), where destruction potentially reduces anatomical and physiological functions, and disfigurements are more apparent [5,6]. This is why the early detection of BCC is critical for its successful treatment and a reduced morbidity [7]. Therefore, BCCs are differentiated into two main categories: lower-risk histologic subtypes (HST nodular, superficial, and pigmented HSTs [8,9,10]) and higher-risk HSTs, including morpheaform (sclerodermiform), infiltrative, micronodular, and basosquamous HSTs [10,11,12,13].

The treatment depends mainly on the HST [14,15]. Superficial and nodular HSTs behave more indolently, while infiltrative, micronodular, and morpheaform HSTs are aggressive and show a greater subclinical extension and recurrence risk [5]. The standard treatment for BCC is surgical removal [16]. Low-risk superficial HSTs can be effectively treated with local destruction (curettage, electrocautery, cryotherapy, or laser ablation) or topical therapies (5% imiquimod and 5% fluorouracil) [14]. For superficial and thin HSTs, we can apply photodynamic therapy (PDT), given that the photosensitizers used in PDT have a limited absorption depth. However, it is important to note that PDT may be also less effective in treating thicker lesions due to the restricted depth of its light penetration [17,18]. Cryotherapy, on the other hand, is a versatile technique suitable for a range of tumor thicknesses, as the depth of the cold penetration achieved is related to the duration of exposure to the cryogen. Therefore, cryosurgery is not limited exclusively to the treatment of thin tumors, but can be adapted to various tumor thicknesses based on the specific requirements of the case [19]. Radiotherapy is not as effective a treatment as surgery [20], but it can be a good alternative for elderly patients [14] and has a lower recurrence rate than cryotherapy [21]. Among surgical methods, Mohs micrographic surgery (MMS) has the best aesthetic outcomes and offers the lowest recurrence rate [22]. Nevertheless, MMS is a time-consuming and specialized procedure. An accurate early evaluation of BCC risk may lead to better care, since many low-risk BCCs could be amenable to immediate destructive treatment, while high-risk BCCs benefit substantially from time-consuming and costly MMS.

Noninvasive imaging techniques have revolutionized BCC diagnosis. These include dermoscopy [23], high-frequency ultrasound (HFUS) [24], optical coherence tomography (OCT) [25], and reflectance confocal microscopy (RCM) [26].

Dermoscopy, a noninvasive technique, has revolutionized the diagnosis of skin lesions by providing enhanced visualization of cutaneous structures that are not discernible to the naked eye, thereby significantly improving the accuracy of dermatologic examinations [27]. In the context of BCC, several algorithms have been developed, utilizing dermoscopy to aid in its diagnosis [28,29,30]. These algorithms primarily rely on the identification of specific dermoscopic features, including vascular structures such as arborizing vessels, pigmented structures like blue-gray ovoid nests, and the presence of ulceration. Additionally, the absence of structures associated with melanocytic lesions, such as areas of network, further assists in distinguishing BCCs [29]. By incorporating these dermoscopic criteria, clinicians can achieve more precise and reliable diagnoses of BCC, enabling timely intervention and improved patient management.

OCT is a cutting-edge imaging technique that offers both high-resolution and effective tissue penetration. It enables the visualization of various cutaneous structures, encompassing the entire epidermis, a portion of the dermis, and appendages [25]. Advancements in OCT technology have further expanded its applications. Optical Doppler tomography utilizes Doppler sensitivity to assess the treatment of vascular lesions [31], while polarization-sensitive OCT utilizes the polarization information carried by light to identify tissue birefringence [32]. In the context of skin, birefringence primarily stems from the organized arrangement of collagen fibers within the dermis. Recent research using line-field confocal optical coherence tomography (LC-OCT) has shown promise in the early diagnosis and subtype classification of BCC. LC-OCT combines the technical advantages of reflectance confocal microscopy and OCT, allowing for the accurate differentiation between BCC and its clinical imitators. Moreover, LC-OCT criteria have been identified as potential predictors for BCC subtypes. For example, nodular BCC is characterized by a poorly defined dermoepidermal junction and dark ovoid structures (often with bright centers), while superficial BCC often presents with epidermal bulges intruding into the upper part of the dermis, surrounded by a darker rim, and infiltrative BCCs appear as ill-defined, narrow, dark, longish structures in the dermis, surrounded by brighter tissue. These advancements in OCT technology, along with LC-OCT criteria, have the potential to enhance the noninvasive diagnosis and subtype classification of BCC, offering valuable insights for its therapeutic management [33,34].

Reflectance confocal microscopy (RCM) is an advanced optical imaging technique that provides the real-time and noninvasive evaluation of skin lesions in vivo. It offers high-resolution imaging capabilities, allowing for a detailed visualization of tissue structures. Notably, RCM has been employed to characterize the histopathologic features of BCC directly in vivo, and the observed confocal features have demonstrated a strong correlation with histological findings. This highlights the ability of RCM to provide valuable insights into the microscopic characteristics of BCC lesions [26].

HFUS imaging is a noninvasive diagnostic technique that utilizes sound waves with frequencies greater than 20 megahertz (MHz) to produce images of tissue structures [15]. HFUS can be used to detect BCC tumor nests—which are hypoechoic—among hyperechoic collagen fibers [35], delineate tumor margins [24], and can estimate tumor depth [36]. BCC risk is mainly defined by tumor location, size, and histological subtype (HST) [37]. While location and size are evident at diagnosis, establishing the HST currently relies on invasive biopsies [38]. Nodular HSTs are usually nodular-shaped hypoechoic lesions with well-defined margins, including multiple hyperechoic spots and cystic degeneration zones, often presenting with a hyperechoic surface and posterior acoustic shadowing [39]. Superficial HSTs are characterized by crawling, well-defined homogenous hypoechoic lesions. Contrary to nodular HSTs, their surfaces are flat without abnormal keratinization [39]. Pigmented HSTs resemble nodular HSTs: their surfaces are elevated and their shapes are oval/irregular, with a well-defined margin with hyperechoic spots within the lesion [39]. Morpheaform BCC lesions are mainly irregular, hypoechoic-heterogenous lesions, infiltrating the dermis. There is often an increase in the echogenicity around the tumor caused by increased fibrosis and edema [13,40].

This prospective study aimed to assess whether optically guided high-frequency ultrasound (OG-HFUS) imaging can identify BCCs with aggressive HST and potentially aid in early treatment planning. We developed and tested a three-step algorithm to differentiate the low-risk HST group from the high-risk HST group and compared these results to those of dermoscopy.

## 2. Materials and Methods

### 2.1. Inclusion and Exclusion Criteria

The inclusion criteria for this study encompassed obtaining informed consent from the patient and a histological confirmation of BCC performed by expert attending dermatopathologists. The exclusion criteria were as follows: the presence of bleeding tumors; BCC that had previously been biopsied or treated with Hedgehog pathway inhibitors, topical therapies, or surgery; and tumors located in areas with inherent technical challenges for OG-HFUS imaging due to their unique curvatures, such as eyebrows, eyelids, ears, and special regions adjacent to the nose. The inability to utilize OG-HFUS in these anatomically complex regions is primarily attributed to the scanner’s impracticality in cases where it cannot be appropriately positioned on the skin surface due to the pronounced curvature of these areas.

### 2.2. Optically Guided High-Frequency Ultrasound Imaging

We utilized a portable OG-HFUS device (Dermus SkinScanner, Dermus Ltd., Budapest, Hungary) to scan the lesions of our patients. For a thorough evaluation of the lesions, we captured at least five cross-sectional images of the examined lesions. In the case of larger lesions, cross-sectional images were taken from each representative part. In addition to the ultrasound imaging capabilities, the device also captured optical images. This OG-HFUS device featured a single-element ultrasound transducer with a 33 MHz nominal center frequency. The resulting ultrasound image was displayed using a color scale, enhancing contrast. The optical image provided a field of view measuring 15 mm × 15 mm, while the ultrasound image extended 12 mm laterally and reached a maximum penetration depth of 10 mm. With an image acquisition time of two seconds, both the optical and ultrasound images were saved and stored in the cloud [41].

### 2.3. The Evaluation of Macroscopic Clinical and Dermoscopic Images

Clinical macroscopic and dermoscopic (non-polarized Heine Delta 20T, Heine Optotechnik GmbH, Herrsching, Germany) photographs of each lesion were collected. To establish the clinical ground truth, we had a panel of ten board-certified dermatologists, who routinely used dermoscopy in their clinical practice, review the clinical and dermoscopic images of the BCCs. They assessed the features of the BCCs and categorized each tumor based on the perceived clinically most relevant histological type and risk category. The four options provided for the assessors were superficial HST (low-risk), nodular HST (low-risk), infiltrative HST (high-risk), and morpheaform HST (high-risk). After an anonymous evaluation, the results of the store and forward image-based clinical evaluations were summarized. We sorted the answers into high-risk (HR) and low-risk (LR). We used contingency tables to count the sensitivity, specificity, positive and negative predictive values, and hit rate. The most relevant histological subtype for each BCC determined by the dermatopathologists was used as the gold standard. In the case of mixed histological features, if high-risk components were present, the lesion was determined to be infiltrative or micronodular BCC, as applicable. In the case of a superficial BCC with a nodular component, the lesion was categorized as nodular BCC.

## 3. Results

### 3.1. Patient Data

Altogether, we had 75 patients, 60 BCC patients to analyze with 63 BCCs (3 patients had 2 BCCs in their bodies) and 15 patients to verify the dataset, with a mean age of 72.9 ± 11.2 years. In total, 60 patients with 63 BCCs were enrolled, with a mean age of 73.1 ± 10.6 years, 34 males and 26 females. A total of 63 lesions were examined. Among the 63 lesions enrolled, 20 were located on the torso, 12 on the cheek, 12 on the nose, 11 on the forehead, 5 on the ear, and 3 on the extremities. The histology showed that 16 lesions had aggressive HST, high-risk (11 with infiltrative and 5 with micronodular histological areas), and 47 had low-risk HST (12 with only superficial and 35 with only nodular or mixed superficial and nodular HST). We verified the algorithm using 15 BCCs. Among the verification dataset, we had 9 nodular, 3 superficial, 1 micronodular, 1 infiltrative, and 1 additional morpheiform BCCs. The mean age was 72.3 ± 13.8 years, with 5 females and 10 males.

### 3.2. Statistical Analysis

For the categorical data and associations, we applied a one-sided Fisher’s exact test. To assess the diagnostic accuracy, a Receiver Operating Characteristic (ROC) curve analysis was employed. Additionally, descriptive statistics were used to calculate the mean age of the study population.

### 3.3. OG-HFUS Imaging

**Figure 1:**
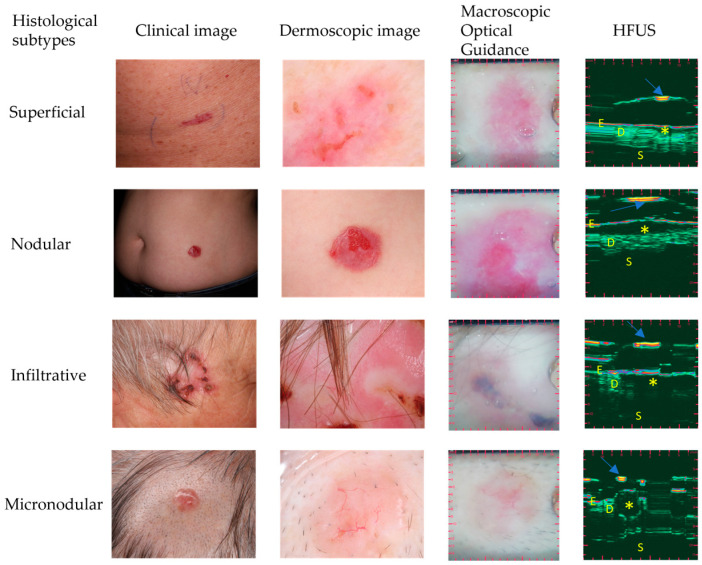
Comparison of different histological subtypes with their clinical image, dermoscopic image, macroscopic optical guidance, and optically guided high-frequency ultrasonography. The first row contains images of a 73-year-old male patient with a superficial BCC. The second row is of a 77-year-old female patient with a nodular BCC. The third row is a 77-year-old male patient with an infiltrative BCC. The fourth row is a 77-year-old female patient with a micronodular BCC. Asterisks (*) represent the tumor and arrows indicate the membrane. E: epidermis, D: dermis, S: subcutis. HFUS: high-frequency ultrasound.

**Figure 2:**
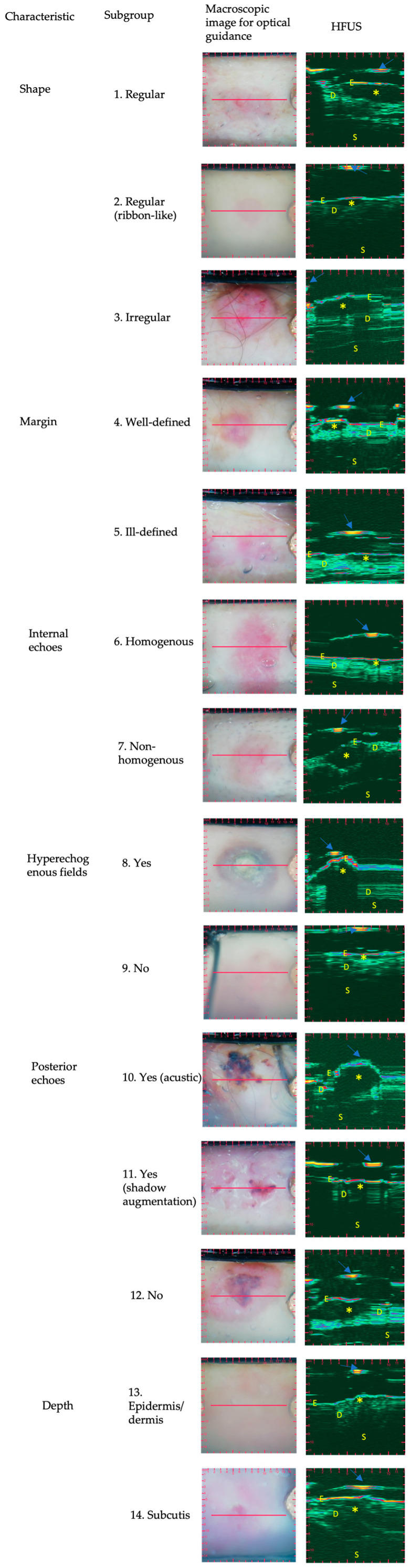
Ultrasound images of BCC histological subtypes: optically guided high-frequency ultrasound characteristics. Basal cell carcinoma subtypes’ ultrasound images of different BCCs subdivided by the optically guided high-frequency ultrasound characteristics, including shape (row 1–3), margin (row 4–5), internal echoes (row 6–7), hyperechoic fields (row 8–9), posterior echoes (row 10–12), and depth (row 13–14). Asterisks (*) represent the tumor and arrows indicate the membrane. E: epidermis, D: dermis, S: subcutis. HFUS: high-frequency ultrasound.

**Figure 3:**
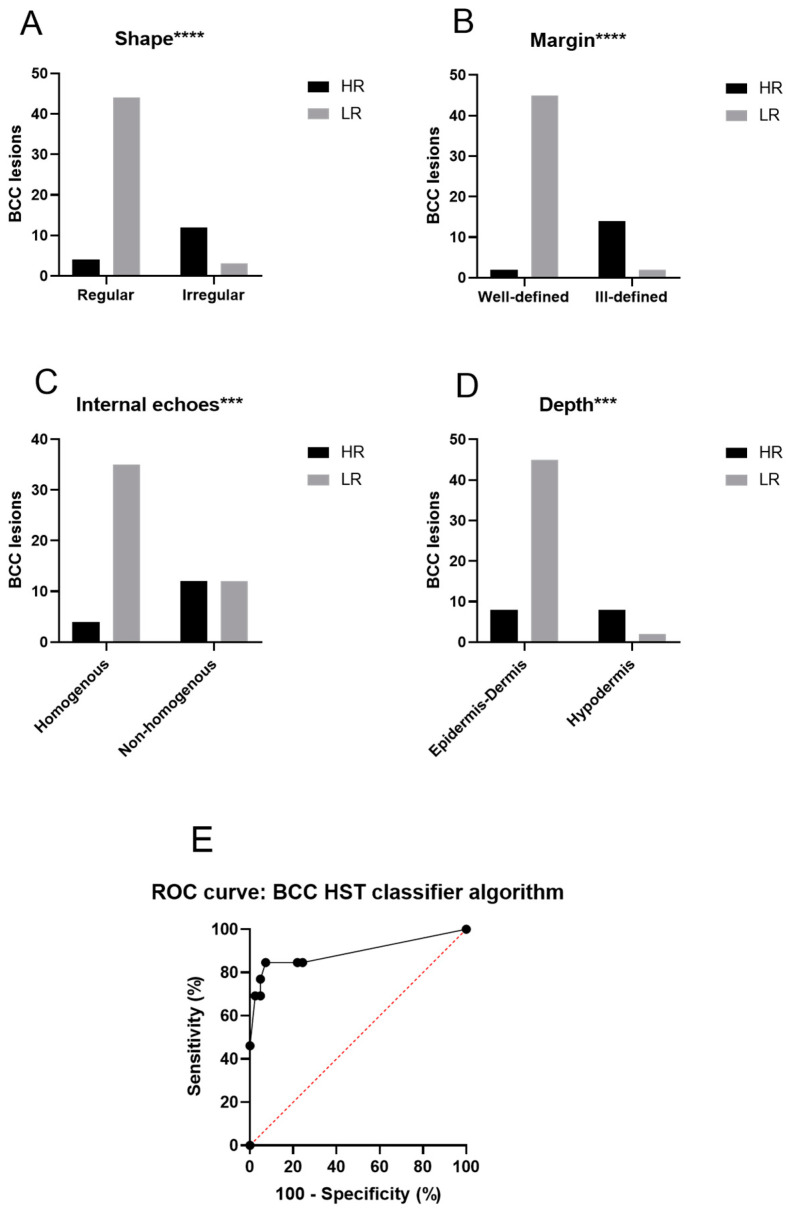
Illustration of the distinct characteristics of the optically guided high-frequency ultrasound images. (**A**) Shape, (**B**) margin, (**C**) internal echoes, (**D**) depth, and (**E**) ROC curve representing the performance of the BCC HST classifier. (**A**–**D**) Show the number of lesions in different subgroups compared to each other using Fisher’s exact test. The subgroups were significantly different regarding the shape (*p* < 0.0001), the margin (*p* < 0.0001), the internal echoes (*p* = 0.0006), and the depth (*p* < 0.0001). Due to the inability to adequately comment on the variables, in all cases with insufficient data in some columns, the variables ‘hyperechoic fields’ and ‘posterior echoes’ were excluded from the Fisher’s test. Thus, we established the categorizing algorithm using these four characteristics. The algorithm successfully differentiated between low-risk and high-risk subgroups. Fisher’s exact test was employed to compare the groups, with *p*-values below 0.05 being considered statistically significant. Additionally, an ROC analysis was performed to highlight the differences between the low-risk and high-risk groups, as identified by the BCC HST classifier algorithm. The Area Under the Curve (AUC) was calculated as 0.8931 (95% confidence interval, *p* < 0.0001). The Y-axis represents sensitivity, while the X-axis represents 1-specificity based on a sample size of 63 BCCs. BCC basal cell carcinoma; HR: high-risk; LR: low-risk; ROC: receiver operating characteristic, *** (*p* < 0.001), and **** (*p* < 0.0001).

Table 1 shows the distributions of different characteristics in the low-risk and high-risk subgroups, whereas Figure 1 and Figure 2 shows the lesions from each HST. The subgroups were markedly separated in terms of some characteristic traits and showed significant differences between low-risk and high-risk HSTs. These characteristics were the shape (*p* < 0.0001), margin (*p* < 0.0001), internal echoes (*p* = 0.0006), and depth (*p* < 0.0001) (Figure 3). Due to the inability to adequately comment on the variables, in all cases, the variables of ‘hyperechoic fields’ and ‘posterior echoes’ were excluded from the Fisher’s test. Nodular BCC lesions were categorized based on their shape, with 88.57% being oval, 2.86% being ribbon-shaped, and 8.57% being irregular. Most lesions showed well-defined margins (94.29%) and homogenous internal echoes (74.29%), while 97.14% had no hyperechoic spots. Posterior echoes were present in 17.14% of cases. In total, 94.29% of the lesions were in the epidermis or dermis. In the case of superficial BCCs, ribbon-shaped lesions constituted the majority (100%), while oval-shaped and irregular-shaped lesions were absent (0%). The margin definition revealed that superficial BCCs predominantly exhibited well-defined margins (100%) and lacked ill-defined margins (0%). An internal echoes analysis showed that 83.33% of the lesions presented homogenous echoes, while 16.67% displayed non-homogenous echoes. Hyperechoic spots were not observed in superficial BCCs (0%), whereas 100% of lesions did not show these spots. Posterior echoes were absent in superficial BCCs (0%), and all the lesions (100%) were confined to the epidermis or dermis without penetration into the hypodermis. Infiltrative BCC lesions displayed distinct HFUS characteristics: 90.91% were irregular-shaped, 9.09% were oval-shaped, and there were no ribbon-shaped lesions. Margin-wise, 90.91% had ill-defined margins, and 9.09% had well-defined margins. Internal echoes varied, with 90.91% having non-homogenous and 9.09% having homogenous echoes. Hyperechoic spots were absent (0%). Posterior echoes were found in 9.09% of cases. Lesion depth: 27.27% were in the epidermis/dermis, while 72.72% were in the hypodermis. Micronodular BCC presented distinct HFUS features: 40% were irregular-shaped, 40% were oval-shaped, and 20% were ribbon-shaped lesions. Margin-wise, 80% exhibited ill-defined margins, while 20% had well-defined margins. Internal echoes varied, with 60% showing homogenous echoes and 40% displaying non-homogenous echoes. No hyperechoic spots were observed (0%). Posterior echoes were absent (0%), and 100% of lesions lacked them. Regarding depth, 80% of the micronodular BCCs extended into the epidermis or dermis, while 20% penetrated into the hypodermis.

### 3.4. BCC Risk Categorization Algorithm

We developed an algorithm based on the OG-HFUS images to categorize the lesions into low-risk or high-risk HSTs (Table 2). Lesions with three points or more were categorized as aggressive HSTs from low-risk with a high sensitivity (82.4%) and specificity (91.3%), as shown by the receiver operating characteristic (ROC) analysis (Figure 3). We verified this algorithm using 15 independent OG-HFUS BCC images evaluated by two trained examiners who were blinded to the histological and clinical characteristics of the tumors. The verification showed that our examiners could distinguish high-risk tumors from low-risk tumors with a sensitivity of 83.33% and specificity 91.66% using the OG-HFUS algorithm.

### 3.5. OG-HFUS Compared to Clinical and Dermoscopic Image Assessment

To understand whether the OG-HFUS algorithm could provide benefits compared to the current routine practice in differentiating between high-risk and low-risk BCC, we established and compared the performance of the dermoscopic evaluation to that of the OC-HFUS algorithm. The cumulative results of the independent dermoscopic and clinical image assessments by ten board-certified dermatologist evaluators only achieved a low sensitivity (40.1%) and specificity (73.1%), with a PPV of 35.8% and NPV of 76.9%, which were much surpassed by the OG-HFUS algorithm’s sensitivity (82.4%), specificity (91.3%), PPV of 94.7%, and NPV of 78.6%.

## 4. Discussion

BCC histology plays a critical role in guiding treatment decisions [14]. The best therapy options for low-risk and high-risk BCC subgroups differ [14], so a reliable and easily accessible method for distinguishing them before treatment would be of great clinical benefit.

Most BCC HFUS research focuses on tumor size and presurgical margin determination, while few projects have assessed the differences between BCC subtype patterns [24,36,42,43]. Notably, Wortsman et al. (2015) conducted a study investigating the correlation between presurgical ultrasound findings and the HSTs of primary BCC tumors using HFUS [44,45]. They visualized and quantified intratumoral hyperechoic spots in 31 patients with histologically proven BCCs, and they achieved a sensitivity of 79% and specificity of 53% for predicting high-risk recurrence subtypes [44,45]. Wang et al. used HFUS to differentiate between invasive and non-invasive BCC subtypes and could distinguish them with an accuracy of 84.0%, supported by a 76.7% validation accuracy [46]. Moreover, Hernández-Ibánez et al. used HFUS to differentiate between different BCC subtypes, and they reached a sensitivity (74.5%) and specificity (73%) which were very close to the incisional biopsy accuracy (sensitivity, 76%; specificity, 82%) [42]. Our study could reach a higher sensitivity (82.4%) and specificity (91.3%); however, our algorithm focused on differentiating high-risk and low-risk subgroups and not on differentiating individual subtypes.

Different descriptions of the HFUS characteristics of BCC subtypes have been published. In a systematic review about BCC HFUS characteristics [35], BCCs could be characterized by hypoechoic tumor masses with hyperechoic spots or hyperechoic areas. The presence of hyperechoic spots played a significant role in categorizing HSTs by severity and identifying specific HSTs, which was also an important characteristic in our BCC HST classifier algorithm. Nodular HSTs were characterized by the presence of more than three hyperechoic spots located centrally and in the periphery. In comparison, a cutoff of more than seven hyperechoic spots was used to identify HSTs with a high risk of recurrence [47,48]. In a retrospective study, Siskou et al. analyzed 100 BCCs of 50 patients to differentiate BCC subtypes from each other (superficial, nodular, micronodular, and infiltrative), focusing mainly on shape. Similar to our results, they found that the infiltrative tumors (*n* = 16/21, 76.2%) were irregular-shaped, while the rest were round (23.8%). Most of the superficial tumors they studied (*n* = 25/29, 86.2%) were ribbon-shaped, and the rest were round (13.8%). Their nodular BCCs (*n* = 26/33, 78.8%) were mostly round-shaped under HST, while the rest were irregular (21.2%), and all of their micronodular tumors (*n* = 2/2, 100%) were round-shaped [49]. This aligns with our studies. All the superficial BCCs were ribbon-shaped (*n* = 12/12, 100%), and almost all of our nodular BCCs were regular (*n* = 31/35, 88.57%)-shaped. The micronodular BCCs in our study were more heterogenous regarding shape, including regular and irregular ones, compared to Siskou et al., where all the micronodular ones were round [49]. A limitation of this study and our study is the lack of morpheaform BCCs. We had one in the validation dataset as a high-risk lesion, but it is a relatively rare form compared to the others and hard to categorize. There was also a discrepancy during the algorithmic evaluation; one of our validators sorted it as a low-risk lesion, while one sorted it as a high-risk lesion.

HFUS provides a detailed visualization of tumor boundaries and their relationship with surrounding tissues. BCCs often exhibit distinct margins, helping determine if the lesion’s margin is well-defined or poorly defined. Well-defined margins suggest a less aggressive HST, while poorly defined margins may indicate a more invasive pattern. Notably, Qin et al. found a significant proportion of BCCs with ill-defined margins (48.1%), which contrasts with the majority being categorized as low-risk HSTs (87%) [50]. Siskou et al. found no association between tumor margin and HST (*p* > 0.005) [49]. In contrast, Khlebnikova et al.’s findings aligned with ours. They investigated the ultrasonographic features of superficial and nodular HSTs, revealing apparent differences in their contour, structure, and margins [47].

In another study by Alfageme et al., 31 BCCs were examined. No significant differences were found between infiltrative and non-infiltrative BCCs regarding their size, hyperechoic dots, vascularization, or strain ratios. However, infiltrative BCCs exhibited an increased marginal stiffness (88.0% versus 18.8%) [51]. While Alfageme’s work highlighted the potential significance of an increased marginal stiffness as an indicator of infiltrative BCCs, with a sensitivity of 89% and specificity of 82%, they reported a PPV of 67% for infiltrative BCCs and an NPV of 95% for non-infiltrative BCCs. This suggests that a heightened marginal stiffness could potentially aid in identifying high-risk HSTs [51]. In our own study, our focus was distinguishing between high- and low-risk BCC HSTs. To accomplish this, we employed OG-HFUS imaging and evaluated the previously described key features, including an irregular shape, ill-defined margins, and non-homogeneous internal echoes, to establish and validate an algorithm that can differentiate between high- and low-risk BCCs.

In another study by Wang et al., BCC’s high-frequency ultrasound features were examined in relation to their histological recurrence risk, using 50- and 20-MHz probes [52]. High-risk HSTs showed a tendency to have irregular shapes compared to low-risk lesions [46]. Both high-risk and low-risk HSTs exhibited hyperechoic spots [52]. This aligns with our findings, and these characteristics contributed to our decision making in creating the BCC HST classifier algorithm. In our observation of the OG-HFUS images, we noted that irregular shapes and ill-defined margins were the most prevalent among the high-risk HSTs.

Our study has certain limitations that need to be acknowledged. These limitations encompass several aspects. Firstly, the study is limited by the low number of high-risk HST BCCs in our dataset, which may have affected the generalizability of our findings to high-risk cases. Additionally, the validation dataset used in this study was relatively small, which could have influenced the robustness of our results when applied to broader populations. Furthermore, we categorized the lesions into two primary depth categories, distinguishing between epidermis/dermis and dermal involvement. While this classification offers a useful initial framework, it may not have captured the full extent of the invasion depth, thus necessitating further investigation. To obtain more precise data on the invasion depth of BCCs, a prospective study comparing the depths of tumors observed using OG-HFUS to histological findings would be valuable, as it could provide a more detailed and accurate assessment of tumor depth [53]. Moreover, Fisher’s exact test was not usable for distinguishing low-risk and high-risk groups based on hyperechoic fields and posterior echo characteristics due to the zero number of cases in certain cells of our contingency tables (Table 1). While this constraint did not impact our algorithm, which utilized the other four characteristics, namely shape, echoes, internal margins, and depth, it is essential to acknowledge the limitations stemming from the small patient cohort in the verification phase of our study. The total number of patients in this phase could potentially have impacted the generalizability and statistical power of our findings. Additionally, OG-HFUS imaging may not be suitable for areas with unique curvatures, such as eyebrows, eyelids, ears, and special regions adjacent to the nose. This constraint further emphasizes the need to consider the applicability and limitations of OG-HFUS in various anatomical regions.

In a real-life clinical setting, lesions with a mixed HST can be treated with respect to the specific individual subtypes within the lesion. For instance, if a lesion primarily presents as superficial and contains a micronodular component, it is possible to surgically excise the micronodular part while employing the standard therapy for superficial BCC on the remainder of the lesion. However, in our study, we classified lesions with high-risk components as high risk, even in mixed lesions, based on the prominent HFUS features to aid in the classification and algorithm development. We acknowledge that this approach, though necessary for the study’s methodology, may not have fully captured the nuanced management of mixed lesions in practice. It is indeed a limitation of the HFUS classification, and we recognize the importance of individualized treatment decisions for mixed histological BCCs in clinical scenarios.

## 5. Conclusions

In summary, our study demonstrated that core OG-HFUS features—shape, margin, internal echoes, and depth—can establish a scoring system for identifying high-risk HSTs, and the superior sensitivity and specificity of OG-HFUS in distinguishing high- and low-risk BCC HSTs when compared to a combined dermoscopic and macroscopic assessment. This heightened precision in early risk assessment carries significant implications for BCC management, particularly in facilitating early minimally invasive treatments for low-risk HSTs. Our findings suggested that OG-HFUS holds the potential to complement other contemporary diagnostic imaging techniques like RCM, revealing risk-correlated features at tissue depths not accessible with the tools that provide a higher resolution in in vivo histological assessments [54]. While our study provides valuable insights, further studies involving larger patient cohorts are imperative to fully explore OG-HFUS’s clinical utility in BCC diagnosis and management. Additionally, studies combining OG-HFUS with higher-resolution imaging are pivotal for catalyzing a paradigm shift in cutaneous oncology.

## Figures and Tables

**Table 1:** Basal cell carcinoma (BCC) low-risk and high-risk subgroups showing the distribution of different optically guided high-frequency ultrasound characteristics, including shape, margin, internal echoes, hyperechoic fields, posterior echoes, and depth (*n* = 63 BCCs).

		Low-Risk HST		High-Risk HST	
Characteristic	Subgroup	Nodular	Superficial	Micronodular	Infiltrative
Shape	Regular (oval)	31	0	2	1
	Regular (ribbon-like)	1	12	1	0
	Irregular	3	0	2	10
Margin	Well-defined	33	12	1	1
	Ill-defined	2	0	4	10
Internal echoes	Homogenous	25	10	3	1
	Non-homogenous	10	2	2	10
Hyperechoic fields	Yes	1	0	0	0
	No	34	12	5	11
Posterior echoes	Yes (acoustic)	5	0	0	0
	Yes (shadow)	1	0	0	1
	No	29	12	5	10
Depth	Epidermis/dermis	33	12	4	4
	Subcutis	2	0	1	7

HST: histological subtype.

**Table 2:** BCC histological subtype classifier algorithm based on the high-frequency ultrasound characteristics. Lesions with 3 or more points are categorized as high-risk lesions, while those with fewer than 3 points remain in the low-risk subgroup.

BCC Histological Subtype Classifier Algorithm Based on Optically Guided High-Frequency Ultrasound Characteristics. 3 or More Points: High-Risk; Below 3 Points: Low-Risk		
Shape	Irregular shape: +3 point	Regular shape: 0 point
Margin	Ill-defined: +3 point	Well-defined: 0 point
Internal echoes	Non-homogenous: +2 point	Homogenous: 0 point
Depth	Hypodermis: +1 point	Epidermis/dermis: 0 point

BCC: basal cell carcinoma.

## Data Availability

The data that support the findings of this study are available from the corresponding author N.K. upon reasonable request.
